# Cascading *cis*-Cleavage on Transcript from *trans*-Acting siRNA-Producing Locus 3

**DOI:** 10.3390/ijms140714689

**Published:** 2013-07-12

**Authors:** Changqing Zhang, Guangping Li, Jin Wang, Shinong Zhu, Hailing Li

**Affiliations:** 1 College of Horticulture, Jinling Institute of Technology, Nanjing 210038, China; E-Mails: zsn@jit.edu.cn (S.Z.); lihailing@jit.edu.cn (H.L.); 2 College of Forest Resources and Environment, Nanjing Forestry University, Nanjing 210037, China; E-Mail: liguangping108@sina.com; 3 State Key Laboratory of Pharmaceutical Biotechnology, School of Life Sciences, Nanjing University, Nanjing 210093, China; E-Mail: jwang@nju.edu.cn

**Keywords:** *trans-*acting siRNA, *cis*-cleavage, ta*-*siRNA-producing locus, miRNA

## Abstract

The production of small RNAs (sRNAs) from phased positions set by microRNA-directed cleavage of *trans*-acting-siRNA-producing locus (TAS) transcript has been characterized extensively; however, the production of sRNAs from non-phased positions remains unknown. We report three *cis*-cleavages that occurred in *TAS3* transcripts in *Vitis vinifera*, by combining high-throughput sRNA deep sequencing information with evolutional conservation and genome-wide RNA degradome analysis. The three *cis*-cleavages can be deciphered to generate an orderly cleavage cascade, and can also produce distinct phasing patterns. Each of the patterns, either upstream or downstream of the *cis*-cleaved position, had a set of sRNAs arranged in 21-nucleotide increments. Part of the cascading *cis*-cleavages was also conserved in *Arabidopsis thaliana*. Our results will enhance the understanding of the production of sRNAs from non-phased positions that are not set by microRNA-directed cleavage.

## 1. Introduction

In plants, many endogenous small RNAs (sRNAs), including microRNAs (miRNAs), heterochromatic small interfering RNAs (siRNAs), natural antisense siRNAs, and *trans-*acting siRNAs (ta-siRNAs), play important roles in regulating gene expression networks [1,2]. The sRNAs are also valuable tools for functional genomics studies. Usually, the sRNAs silence gene expression by either degrading mRNA or repressing translation but, in a few cases, they also generate a population of secondary siRNAs. The ta-siRNAs are secondary siRNAs that are produced by a miRNA-targeted trigger that bridges the pathways of miRNA and siRNA regulation. ta-siRNAs can regulate plant development, metabolism, and responses to biotic and abiotic stresses, and thus have received more attention in the recent decade [3–5].

During the biogenesis of ta-siRNA, a single-stranded RNA is transcribed from a ta-siRNA-producing locus (TAS) and then cleaved by a phase-initiator (a miRNA or, in some cases, a ta-siRNA). Then, RNA-dependent RNA polymerase 6 (RDR6)-dependent conversion of the resulting fragments into double-stranded RNA and its subsequent cleavage by dicer-like 4 (DCL4) at every ~21 nucleotide (nt) relative to the phase-initiator cleavage site generates ~21-nt phased sRNAs. Some of the phased sRNAs become ta-siRNAs by binding argonaute (AGO) proteins to direct a *trans-*cleavage of targeted mRNAs [5–7]. Plant TASs can be classified into at least eight families, based on initiator-dependence, sequence similarity, and target gene identity. *TAS1* and *TAS2* are targets of miR173 and their ta-siRNAs can target the pentatricopeptide repeat genes [8]. *TAS3* is a target of miR390 and its ta-siRNA can target the auxin response factor gene family [8]. The initiator of *TAS4* is miR828, and the *TAS4* ta-siRNA can target the MYB transcription factor gene family [9]. *TAS5* is triggered by miR482 and its ta-siRNA can target the Bs4 resistance gene [10]. miR156 and miR529 initiate *TAS6*, which targets an mRNA that encodes a zinc finger protein [11]. miR828 initiates *TAS7*, which can target 13 genes, including genes that encode the leucine-rich receptor protein kinase-like protein and a calcium-transporting ATPase [12]. *At1g63130* is a pentatricopeptide repeat gene that was reported to be cleaved by *TAS2*-derived ta-siR2140 [13]. *TAS3* is flanked by two miR390 binding sites; one of which can be cleaved by the interaction of miR390 and AGO7, and another that is non-cleavable. Both binding sites are critical for the biogenesis of the *TAS3* ta-siRNAs. In contrast, other TASs have only a single miRNA binding site and are cleaved by the interaction of miRNA/ta-siRNA and AGO1. Recently, AGO2 has been reported to mediate *cis*-cleavage of *TAS1c*, although its slicer activity has not been demonstrated so far [14]. Taken together, it might be expected that many of the sequenced sRNAs could be mapped onto the phased positions set by the phase-initiator. Yet, while many of the sRNAs were successfully mapped, unexpectedly, many were mapped onto non-phased positions; that is, the intervals between the phased positions [12,13]. These sRNAs have been called “non-phased sRNAs” and how they are produced remains unclear.

The recent publication of the degradome library generated from cleaved mRNA fragments and sRNA libraries generated by high-throughput deep sequencing has enabled the study of all the cleavages that occur in a TAS transcript [15,16]. Here, we studied *cis*-cleavage of grapevine *TAS3* and found a cascading *cis*-cleavage, which produce sRNAs from the so-called non-phased positions and broaden the known scope of non-phased sRNA production.

## 2. Results and Discussion

### 2.1. Overview of Small RNA Distribution on *TAS3* from *Vitis vinifera*

Previously we reported that the *TAS3* from *Vitis vinifera* (vvi*TAS3*) can be targeted by vvi-miR390 to trigger ta-siRNA production in grapevine [12]. Here, to determine the distribution of sRNAs on the vvi*TAS3* transcript, a sRNA library from grapevine leaves (GEO: GSM458927) was used. To improve mapping confidence, only the sRNAs that mapped to a single site on the whole *V. vinifera* genome were used, because they could be attributed with certainty to a particular locus.

**Figure 1:**
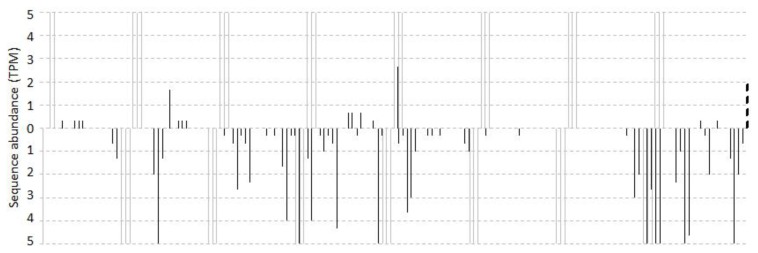
Abundance distribution of sRNAs mapped to vvi*TAS3*. The number of reads with a 5′ end at each position is plotted. Bars above the sequence represent sense reads; those below represent antisense reads. The cleavage site of vvi-miR390 is marked by a vertical dotted line. Vertical gray lines indicate the miRNA-set 21-nt phased cleavage, allowing a 1-nt offset. Regions with TPMs greater than five are not shown.

As a result of the mapping, we detected 131 unique sRNAs, representing 3969 reads, which matched perfectly to vvi*TAS3* (Figure 1). The 5′ ends of the reads occupied 79 positions on the transcript. Only 14 (18%) of the positions were found to belong to phased positions set by vvi-miR390 when a 1-nt offset from the phased positions was allowed. After filtering out sRNAs that had TPM (tags per million) values of five or less, some unique sRNAs that mapped to non-phased positions remained (Figure 1). The percentage of phased sRNA positions increased from 18% to 40%. Together, these results showed that some sRNAs are really generated from non-phased positions, and might even be functional because of the relatively high levels at which they are often expressed.

### 2.2. Computational Prediction and Validation of *cis*-Cleavages

Recent reports have shown that many functional siRNAs belong to a class of 21–22-nt 5′U/A sRNAs [14]. Therefore, we filtered out the potential siRNAs from the mapped sRNAs by limiting the length of the reads to 21 nt and the 5′ end to U/A. As a result, we detected 35 sRNAs that were mapped to the antisense strand that passed the rule.

**Figure 2:**
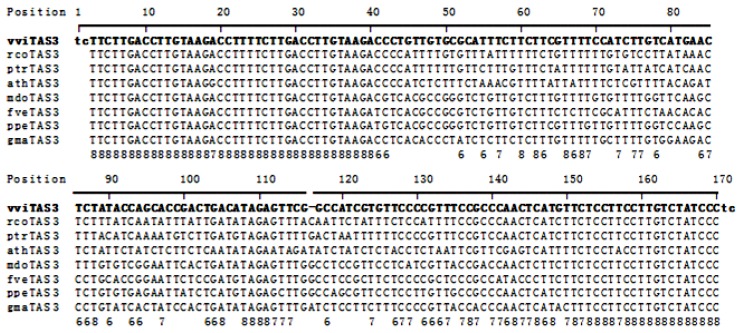
Alignment of *TAS3* DNA sequences from eight dicotyledonous plant species. The numbers in the last row indicate the number of species with the same nucleotide as vvi*TAS3* at each of the marked positions. Only numbers over six are shown. vvi, *Vitis vinifera*; rco, *Ricinus communis*; ptr, *Populus trichocarpa*; ath, *Arabidopsis thaliana*; mdo, *Malus domestica*; fve, *Fragaria vesca*; ppe, *Prunus persica*; gma, *Glycine max*.

Additionally, it has been reported that many functional cleaved positions tend to be conserved through evolution, and they have been found to be highly conserved in alignments of genomic sequences from different species [8]. To identify conserved positions in the vvi*TAS3* transcript, we compiled a dataset of *TAS3* sequences from eight dicotyledonous plants; namely, *V. vinifera*, *Ricinus communis*, *Populus trichocarpa*, *Arabidopsis thaliana*, *Malus domestica*, *Fragaria vesca*, *Prunus persica*, and *Glycine max*, and aligned them using ClustalX2 [17] with the default parameters (Figure 2). The multiple sequence alignment showed that there are no insertions or deletions among the *TAS3* sequences, except for a one-nucleotide deletion in vvi*TAS3* between position 116 and 117. The conserved positions were filtered by requiring each position to be conserved in at least six of the species, and to correspond to the 10^th^ position of the 35 candidate *cis*-acting sRNAs. We found that 19 of the 35 sRNAs passed these rules.

Finally, using a parallel sequencing sRNA library that contained degradome tags from grapevine leaves [18], we validated the predicted *cis*-cleaved positions on the vvi*TAS3* transcript by requiring that the *cis*-cleaved positions overlapped with the 5′ end of the RNA degradation fragment mapped onto the TAS. As a result, three of the positions, 63, 85, and 138, were validated. The corresponding *cis*-acting siRNAs (ca*-*siRNAs) were three 21-nt 5′U ca*-*siRNAs and one 22-nt 5′U ca*-*siRNA (Table 1). It has been reported that the size of the sRNAs and the 5′-terminal nucleotide are critical for the sorting of AGO. AGO1 binds 21-nt 5′U sRNAs, but in some cases, it also binds 22-nt 5′U sRNAs [14]. In Arabidopsis, the 22-nt 5′U 3′D10(−) from *TAS1c* has been reported to mediate its *cis*-cleavage by binding to AGO1, and the 21-nt 5′U 3′D6(−) from *TAS1c* has also been shown to mediate *TAS1c* cleavage by binding to AGO1; however, in this case, the cleaved site is not its original site [14]. In this study, we found three ca*-*siRNAs that were 21-nt 5′U sRNAs and one that was a 22-nt 5′U sRNA. These results implied that the four ca-siRNAs were all loaded to AGO1.

It has been demonstrated that two miR390 binding sites located on each side of *TAS3* are critical for *TAS3* ta-siRNAs biogenesis [8]. Therefore, we looked for ca-siRNA targeting sites on vvi*TAS3* and the flanking 300 bp upstream and downstream of the gene where the two miR390 binding sites were located. We found one targeted site on vvi*TAS3* for each of the ca*-*siRNAs. This finding suggested that *cis*-cleavage might use a different mechanism from the mechanism used by miR390 to initiate the cleavage of vvi*TAS3*.

We used the same method and criteria to examine the antisense strand that was not targeted by miR390. Surprisingly, no ca*-*siRNA targeting sites were detected on the antisense strand of vvi*TAS3*. The asymmetrical distribution between the targeted and non-targeted strand might imply that the non-targeted strand is readily in a double-stranded RNA form and is constantly processed by DCL4 and, therefore, protected from *cis*-cleavage. In addition, it might support the hypothetically biological function of *cis*-cleavage, *i.e.*, inactivating TAS transcription to feedback control ta-siRNA’s production, as the sense strand was a template strand synthesizing antisense strand, so it would be more effective when the *cis*-cleavages preferred to occur in sense strand rather than in antisense strand.

### 2.3. Cascading *cis*-Cleavages

**Figure 3:**
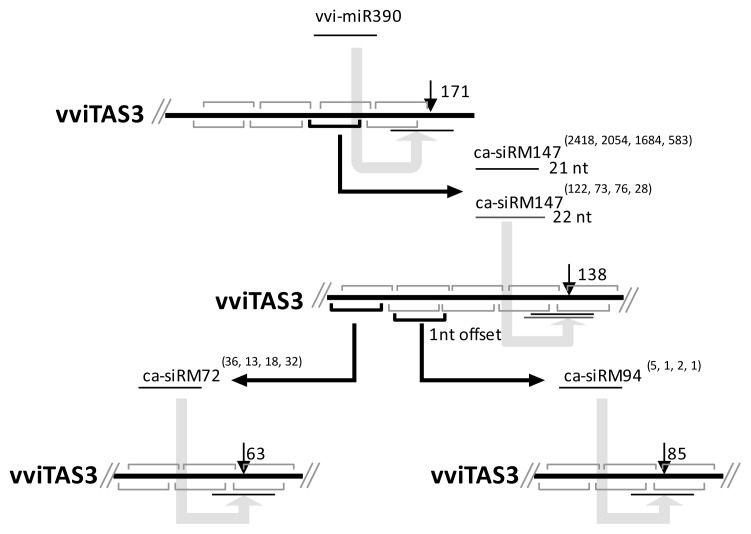
Cleavage cascades generated by *cis*-cleavages of siRNA on the sense strand of vvi*TAS3*. The four numbers in the brackets indicate the raw abundance of ca-siRNA in grapevine berries, leaves, inflorescences, and tendrils, respectively.

After identifying the *cis*-cleaved positions and their ca-siRNAs, we investigated how ca-siRNA production is triggered. It has been shown that ca-siRM147 can be triggered by miR390 [12], but for other ca-siRNAs the triggers remain unclear because they are out of the register set by miR390. To identify possible initiators, we investigated the distribution of *cis*-cleaved position and the locations of ca-siRNA 5′ ends on the vvi*TAS3* transcript. We found that the 5′ end of ca-siRM72 occurred precisely at the register set by ca-siRM147 in which the cleaved site of ca-siRM147 located on sense strand was 65 nt away from the 5′ end of ca-siRM72 on antisense strand. The 5′ end of ca-siRM94 was offset by 1 nt from the register set by ca-siRM147. Because the cleavage of phasing sRNAs often occurs within 1–2 nt of the phased position [8,13], we propose that the production of ca-siRM147, ca-siRM72, and ca-siRM94 is triggered by miR390, ca-siRM147, and ca-siRM147, respectively. The cascading *cis*-cleavage that we have proposed is shown schematically in Figure 3.

### 2.4. The Accumulated Levels of ca-siRNAs

When we analyzed the accumulated levels of the ca-siRNAs in the grapevine leaf, berry (GEO: GSM458930), inflorescence (GEO: GSM458929), and tendril (GEO: GSM458928) libraries, we found that the levels were in agreement with the cleavage cascades. The abundance of the ca-siRNAs that were located upstream of the cascade was always higher than the abundance of the ca-siRNAs that were downstream. For example, in the grapevine leaf library, the 21-nt ca-siRM147 located upstream had 2054 sequenced reads, while ca-siRM72, located downstream of ca-siRM147 cleavage, had only 13 sequenced reads. Moreover, the ca-siRNAs that occurred precisely at the register were always more abundant than those that occurred out of the register. For example, in the grapevine leaf library, ca-siRM72 had 13 sequenced reads, while ca-siRM94, which was shifted by 1 nt from the phased positions set by ca-siRM147, had only one sequenced read. Similar results were obtained for the other three tissues (Figure 3).

### 2.5. *cis*-Cleavages Produced sRNAs in Increments of Approximately 21 nt

To test whether or not *cis*-cleavages can also produce phased sRNAs in increments of approximately 21 nt, we searched for sRNAs with 5′ ends that overlapped the predicted phased and non-phased positions by allowing an offset of 1 nt. As expected, each *cis-*cleavage had a set of corresponding phased sRNAs arranged in ~21-nt increments upstream and downstream of the cleavage position (Table 2).

To test whether or not the phased patterns produced from *cis*-cleavages were statistically significant, we developed an improved equation (see Experimental Section) by modifying previous algorithms [12,13,19] to evaluate the phasing pattern set by *cis-*cleavage. First, the new equation is not constrained by the previous 231-bp length requirement [12,13,19], but requires only a multiple of 21 nt, which provides a more accurate TAS evaluation, especially for TASs that are longer or shorter than 231 bp. Second, our equation uses a variable *s* to reflect the maximum offset from a phase position [12,19], making the evaluation more flexible. This equation could be applied to TAS identification in the future. Using our improved algorithm [12,13,19], we determined that two *cis-*cleavages had significant phasing patterns (*p*-value < 0.01). When the number of sRNAs located in the phased positions set by ca-siRNAs was counted, we found that 54 unique sRNA located in non-phased positions set by miRNA390 were included in the phasing patterns. These results suggested that some common processes might be used for both miRNA-mediated ta-siRNA production and the *cis*-cleavage of siRNA.

### 2.6. The Conservation of ca-siRNAs and Cascading *cis*-Cleavages

To examine the conservation of *cis*-cleavages further, we first looked for the presence of ca-siRNAs in the sRNA datasets of *V. vinifera*, *A. thaliana*, *M. domestica*, and *P. persica* downloaded from the Gene Expression Omnibus (GEO) or the plant MPSS databases. We found that although the accumulation levels of the four ca-siRNAs varied in the different species, they were expressed in all four species, except for 22-nt ca-siRNA147, which was not detected in *P. persica* (peach) (Table 3).

**Figure 4:**
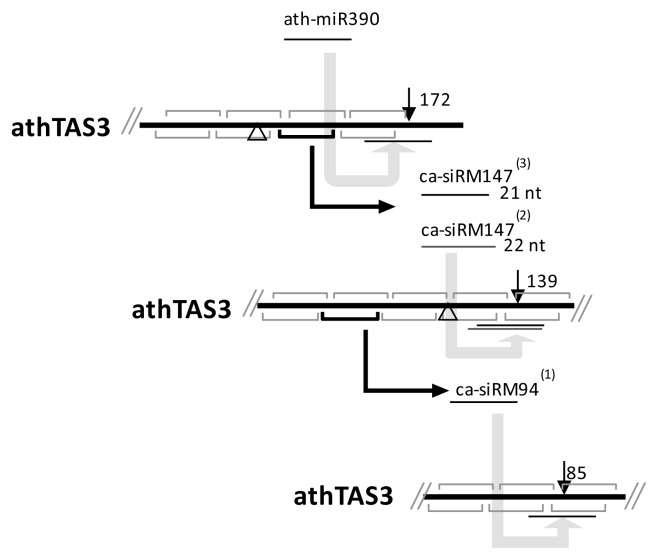
Cleavage cascades generated by *cis*-cleavages of siRNA on the sense strand of ath*TAS3*. The triangle indicates the position of the nucleotide deletion in vviTAS3. The number in the bracket indicates the raw abundance of ca-siRNA in Col7d (GEO: GSE20197).

We then evaluated the corresponding *cis*-cleavages based on the Col7d samples (GEO: GSE20197) and the degradome library (GEO: GSM280227) from Arabidopsis, and found that, two *cis*-cleavages occurred in positions 85 and 139 (equivalent to position 138 in vvi*TAS3* because of the nucleotide deletion in the vvi*TAS3* sequence) were also validated on ath*TAS3* (Figure 4). The accumulated levels of ca-siRNAs in Col7d sample were also in agreement with the cleavage cascades. In which, the 21-nt and 22-nt ca-siRM147 located upstream had, respectively, three and two sequenced reads, while ca-siRM94, located downstream of ca-siRM147 cleavage, had one sequenced reads. In a previous study [5], it was suggested that *cis*-cleavage occurred on position 139 in ath*TAS3*, although the ca-siRNA and functional sRNAs were not found. Here, we detected the ca-siRNA and a secondary ca-siRNA product (ca-siRNA94), which we believe provides enough evidence to establish the *cis*-cleavage on *TAS3*. The findings reported here for ath*TAS3* indicate that cascading *cis-*cleavage is conserved.

## 3. Experimental Section

### 3.1. Sources of sRNA Libraries

In this study, we used four deep sequencing sRNA datasets; namely, two degradome library and two sRNA libraries. All the datasets were downloaded from the Gene Expression Omnibus (GEO). The GEO accession numbers for these libraries are given in the Results section.

### 3.2. Evaluation of Phasing Patterns Set by *cis*-Cleavage

Once a *cis-*cleaved position was determined, the numbers of phased and non-phased positions were counted upstream and downstream of the cleavage sites respectively. Phased positions refer to positions arranged in 21-nt increments relative to the cleavage position as well as to positions shifted by *s* nt relative to the positions of 21-nt increments. Non-phased positions are all the other positions. The *p*-value of each detected phasing pattern was calculated based on a random hyper-geometric distribution using an improved equation based on previously used algorithms [12,13,19,20]:

(1) $$\text{P}({\text{k}}_{1})=\sum_{\text{x}={\text{k}}_{1}}^{\text{min}({\text{k}}_{1}+{\text{k}}_{2},\frac{2\text{L}}{21}-1)}\frac{\left(\begin{array}{c} \left(2\text{L}-1)-(\frac{2\text{L}}{21}-1)\times (2\text{s}-1\right)\\ {\text{k}}_{2} \end{array}\right)\left(\begin{array}{c} \frac{2\text{L}}{21}-1\\ {\text{k}}_{1} \end{array}\right)}{\left(\begin{array}{c} (2\text{L}-1)-(\frac{2\text{L}}{21}-1)\times 2\text{s}\\ {\text{k}}_{1}+{\text{k}}_{2} \end{array}\right)}$$

Where *L* is the length of the detected pattern and is a multiple of 21, *K*_1_ is the number of phased positions having sRNA hits, *K*_2_ is the number of non-phased positions having sRNA hits, and *s* is the maximum allowed offset from the phase position.

### 3.3. Expressional Conservation of ca-siRNAs

The expressional conservation of the grapevine ca*-*siRNAs was investigated by performing a search against 84 sRNA libraries from grapevine, apple, Arabidopsis, and peach. The sRNA libraries of grapevine (GEO: GSE18405) and apple (GEO: GSE36065) were downloaded from the GEO and the sRNA libraries of Arabidopsis and peach were used from the MPSS databases [21]. The normalized abundance is the raw expression value divided by the total number of signatures and multiplied by 1,000,000.

## 4. Conclusions

In this work, we reexamined the distribution of sRNAs on vvi*TAS3* using a stringent threshold that used only the sRNAs that mapped to a single site on the whole *V. vinifera* genome and that had normalized abundant values of one or more TPM. Our results showed that the non-phased positions were indeed located by some of the uniquely mapped sRNAs. We identified three *cis*-cleavages that directed by four ca-siRNAs at positions 63, 85, and 138 on vvi*TAS3* by combining computational predictions and validation. We found that three *cis*-cleavages, together with their ca-siRNAs, formed a cascading *cis*-cleavage. The accumulated levels of four ca-siRNAs in the berry, leaf, inflorescence, and tendril libraries of *V. vinifera* also agreed with the cascade. A comparative analysis showed that the expression levels of the four ca-siRNAs were conserved among grapevine, apple, peach, and Arabidopsis, and part of the *cis*-cascade was also identified in Arabidopsis. We also found that sRNAs were located at the phased positions set by ca-siRNA. These results broaden the known scope of non-phased sRNA production. We also developed an improved equation by modifying previous algorithms to evaluate the phasing pattern set by *cis*-cleavage. It could be applied to TAS identification in the future.

## Figures and Tables

**Table 1:** Validated *cis*-cleaved positions on vvi*TAS3* transcript and their ca-siRNAs.

*cis*-cleaved position	ca-siRNA	
	Name	Sequence
63	ca*-*siRM72	UGGAAAACGAAGAAGAAAUGC
85	ca*-*siRM94	UGGUAUAGAGUUCAUGACAAG
138	ca*-*siRM147	UGAGUUGGGCGGAAACGGGGA UGAGUUGGGCGGAAACGGGGAA

**Table 2:** Phased patterns set by different *cis*-cleavages.

*cis*-cleaved position	Upstream of *cis-*cleaved position			Downstream of *cis-*cleaved position		
	
	Number of phased sRNAs	Number of non-phased sRNAs	*p* value *	Number of phased sRNAs	Number of non-phased sRNAs	*p*-value
63	2	12	1.11 × 10–1	3	47	4.92 × 10–1
85	5	27	**7.54 × 10–3**	4	30	1.00 × 10–1
138	8	41	**4.71 × 10–4**	3	16	7.43 × 10–2

* *p*-values less than 0.01 are in bold font.

**Table 3:** Conservation of ca-siRNAs in the sRNA datasets of four species.

ca-siRNAs		Average of normalized abundance (TPM)			
	
Name	Length	Grapevine	Apple	Peach	Arabidopsis
ca*-*siRM72	21 nt	6	2	1	1
ca*-*siRM94	21 nt	1	3	14	1
ca*-*siRM147	2 1nt	419	32	9	2
	22 nt	18	1	0	2
